# The Therapeutic Landscape of Salivary Gland Malignancies—Where Are We Now?

**DOI:** 10.3390/ijms232314891

**Published:** 2022-11-28

**Authors:** Robbert Cleymaet, Tijl Vermassen, Renaat Coopman, Hubert Vermeersch, Stijn De Keukeleire, Sylvie Rottey

**Affiliations:** 1 Department of Oromaxillofacial and Plastic Surgery, Ghent University Hospital, 9000 Ghent, Belgium; 2 Department Medical Oncology, University Hospital Ghent, 9000 Ghent, Belgium; 3 Department Basic and Applied Medical Sciences, Ghent University, 9000 Ghent, Belgium; 4 Cancer Research Institute Ghent, 9000 Ghent, Belgium; 5 Department Internal Medicine, University Hospital Brussels, 1090 Brussels, Belgium; 6 Drug Research Unit Ghent, University Hospital Ghent, 9000 Ghent, Belgium

**Keywords:** salivary gland malignancies, diagnostics, targeted therapy, personalized medicine, molecular pathways

## Abstract

Salivary gland malignancies (SGMs) account for less than 5% of new diagnoses in head and neck tumors. If feasible, surgery is the preferred treatment modality. Nevertheless, some malignancies have a tendency of recurrence, with possible distant metastasis. Alternative treatment strategies, such as primary radiation or chemotherapeutics, often present low response rates. As a result, there is an unmet need for novel therapeutic approaches. Nowadays, target-based therapies (e.g., small inhibitors and immunotherapy) are used by the medical oncologist for possible treatment of advanced SGMs. Based on recent published trials, some novel treatments may provide additional disease control for some patients. However, sample sizes are small, the general findings are unsatisfactory, and a lot of uncertainties remain to be elucidated. Nevertheless, research shows that patients do not benefit from blind administration of systemic treatments and therefore a more personalized approach is highly needed. The aim of this review paper is to summarize the most recent advances in the biological understanding and molecular pathways of salivary gland cancers, the association of these pathways with the current treatments used and their implications for more personalized targeted-based therapies.

## 1. Introduction

Salivary gland malignancies (SGMs) are rare malignancies in the head and neck region representing fewer than 5% of the newly diagnosed head and neck neoplasms [1,2]. The WHO describes almost 40 different tumors in the salivary gland region of which the most frequent malignant SGMs are muco-epidermoid carcinoma (MEC) [3,4,5,6,7,8], adenoid cystic carcinoma (AdCC) [5,9,10,11,12], salivary duct adenocarcinoma (SDC) [5,13,14,15] and acinic cell carcinoma (AcCC) [5,16,17,18,19] (Table 1).

Swelling at the salivary gland’s location is often the first clinical symptom indicative of a tumoral process, as well as peripheral facial nerve loss, ingrowth and fixation at the skin or the presence of lymph nodes in the neck region. Preliminary diagnosis is routinely done via ultra-sound guided fine needle aspiration cytology (FNAC). However, its use is limited when it comes to identifying the subtype and grade of the malignant tumor [20].

**Table 1:** Overview of the most common SGMs.

Subtype	Prevalence	Treatment	Prognosis
MEC	29–35% [3,4,6]	- Surgery in case of resectable tumor - Adjuvant radiotherapy in case of perineural invasion, lymph node involvement, advanced high-grade tumors, positive margins after resection and extra-glandular extension [8]	Depends on grading 5-year OS = 79.3% 5-year DFS = 76.5% [7]
AdCC	20–22% [9,10,11]	- Complete surgical resection - Postoperative radiotherapy is almost always used for AdCC [12]	5-year OS = 55–98% 15-year OS = 23–40% [11]
SDC	5–10% [13,14]	- Surgery and radiation therapy [13]	5-year OS = 30–48% [15]
AcCC	10–25% [16,17,18,19]	- Complete surgical removal via total or subtotal parotidectomy - Postoperative radiotherapy in all cases except T1N0 or T2N0 [16]	5-year OS = 91% 10-year OS = 88% [16]

AcCC, acinic cell carcinoma AdCC, adenoid cystic carcinoma; DFS, disease-free survival; MEC, mucoepidermoid carcinoma; OS, overall survival; SDC, salivary duct carcinoma; SGMs, salivary gland malignancies.

Ultrasound-guided core needle biopsy provides a more precise preoperative diagnosis, mostly because the technique obtains larger tissue samples, preserves the histologic architecture, and can thereby provide a more accurate insight in tumor typing, grading and immunohistochemistry [21]. Initial diagnosis is often supplemented with a computed tomography (CT) scan or magnetic resonance imaging (MRI). Importantly, these imaging techniques can be leveraged to evaluate local extent and perineural extension, both of which are critical for a correct TNM staging [22]. Novel MRI techniques, including dynamic contrast-enhanced MRI and diffusion-weighted MRI, are being investigated to differentiate between benign and malignant processes [23]. Finally, to detect tumor extension, local recurrence, nodal involvement, and distant metastasis, positron emission tomography CT (PET-CT) is more accurate than classical CT [24]. However, PET-CT is not useful in classifying SGMs as benign or malignant [25]. Although great improvements in imaging have been reported in recent years, it remains difficult to fully rely on imaging for the final SGM diagnosis, thus leaving biopsy as the gold standard.

Upon conclusive diagnosis, a broad local excision with negative margins is the first-line curative therapy for SGMs in the absence of distant metastases. This can be combined with concurrent/adjuvant radiotherapy and/or chemotherapy, depending on lymph node involvement, recurrence, or metastasis of the SGM. The treatment strategies for localized SGMs have extensively been described in other reviews and will not be covered in the current review [26,27,28,29].

Once the patient is diagnosed with locally advanced, inoperable recurrent, or metastatic disease, systemic treatment is mostly the therapy of choice. Classic chemotherapeutic agents have proven only limited durable response. Thus, the necessity has arisen of implementing targeted molecular agents into the armamentarium. However, clinical trials introducing novel treatment strategies in SGMs are scanty [30].

In the current review paper, we will provide an extensive overview on the available systemic treatment regimens in patients with advanced salivary gland carcinoma. In addition, we elaborate on the molecular pathways involved in the carcinogenesis of SGMs and the consequent importance of more personalized and targeted-based therapies. The latter will be illustrated by introducing a case-report from our university hospital.

## 2. Systemic Therapies

### 2.1. Chemotherapy

Prior to the advent of targeted therapies, only chemotherapy was available for as palliative treatment in inoperable and advanced/metastatic malignancies of the salivary gland [31]. Various chemotherapy regimens, both in monotherapy or as combination, have been tested in salivary gland carcinoma with objective response rates (ORR) ranging between 0% and 70% [32,33,34,35,36,37,38,39,40,41,42,43,44,45,46,47,48,49,50,51,52,53,54]. An overview of the chemotherapeutic options tested in SGMs is given in Table 2. However, there are presently no conventional chemotherapeutic options recommended in the (neo-)adjuvant nor metastatic setting.

### 2.2. Target-Based Therapy

**Figure 1:**
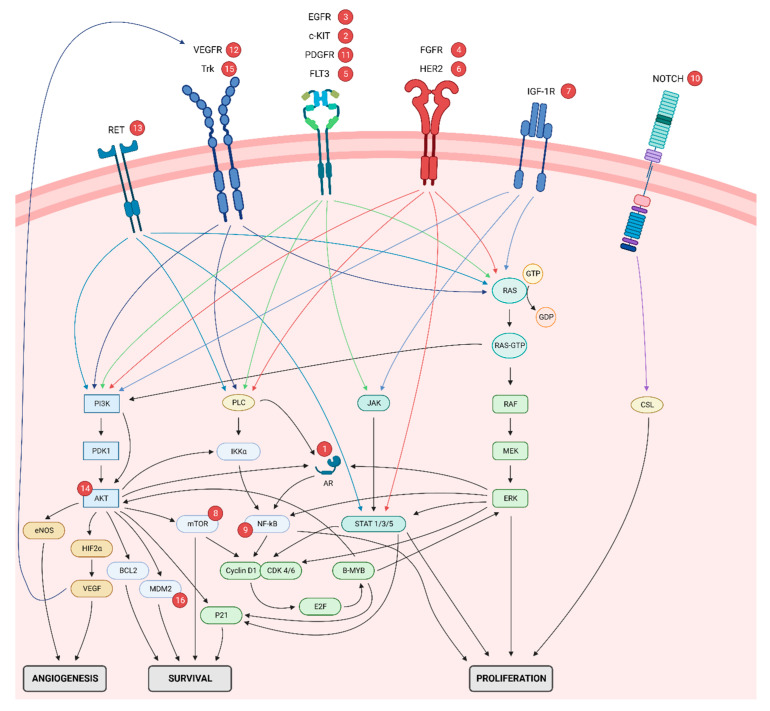
**Overview of receptors and molecular pathways involved in SGMs.** Illustration of the receptors and intracellular pathways that can initiate angiogenesis, proliferation, and survival in SGMs. Red spheres indicate targeted therapies which have been developed for numerous of these receptors and intracellular molecules: **1**: abiraterone, bicalutamide, enzalutamide, leuprorelin; **2**: apatinib, axitinib, dasatinib, dovotinib, imatinib, lenvatinib, sorafenib, sunitinib; **3**: cetuximab, dacomitinib, gefitinib, lapatinib; **4**: dovotinib, nintedanib, lenvatinib, regorafenib; **5**: cabozantinib, dovotinib, sorafenib, sunitinib; **6**: lapatinib, pertuzumab, trastazumab; **7**: figitumumab, R1507; **8**: everolimus; **9**: bortezomib; **10**: AL101, BMS-986115, brontictuzumab, CB-103, crenigacestat; **11**: axitinib, dovotinib, imatinib, lenvatinib, nintedanib, regorafenib, sorafenib, sunitinib; **12**: apatinib, axitinib, cabozantinib, dovotinib, lenvatinib, nintedanib, regorafenib, sorafenib, sunitinib; **13**: apatinib, cabozantinib, dovotinib, lenvatinib, sorafenib, sunitinib; **14**: nelfinavir; **15**: VMD-928; and **16**: APG-115. It is clear that SGMs have a multitude of pathways which can sustain carcinogenesis and therefore provide the carcinoma with an excellent tool for drug resistance to a specific targeted therapy. AR, androgen receptor; EGFR, epidermal growth factor receptor; FGFR, fibroblast growth factor receptor; FLT3, fms-like tyrosine kinase 3; HER, human epidermal growth factor receptor; IGF1R, insulin-like growth factor 1 receptor; mTOR, mammalian target of rapamycin; NF-κB, nuclear factor-κB; NOTCH, Neurogenic locus notch homolog protein; PDGFR, platelet-derived growth factor receptor; RET, rearranged during transfection; SGMs, salivary gland malignancies; Trk, tropomyosin receptor kinase; VEGFR, vascular endothelial growth factor; VEGFR, vascular endothelial growth factor receptor.

Nowadays, researchers are attempting to fully elucidate the molecular etiology of SGMs. Angiogenesis, proliferation, and survival are three hallmarks of carcinogenesis. To enable the tumor to grow and survive, multiple receptors and pathways, such as PI3K, PLC, JAK, and RAS pathways; are activated or overexpressed. This is also the case for SGMs (Figure 1).

Targeting specific receptors or intracellular pathways may therefore be the way to go for SGMs, due to the numerous subtype-specific genomic alterations and upregulations. Nevertheless, molecular variation among different types of SGMs also complicates the selection of the appropriate drug according to a review paper from Sahara et al. [55]. Below, we summarize which receptors, intracellular molecules, and epigenetic factors play an important role in SGMs, and which targeted therapies are currently available to inhibit the aberrant expression/alteration in SGMs.

#### 2.2.1. Receptors

Several overexpressing receptors, e.g., receptor tyrosine kinases, have been indicated for SGMs, especially AdCC. Overexpression of c-KIT has been identified as a hallmark of AdCC histology as 80–90% of cases of AdCC overexpress c-KIT [56]. VEGF, and consequently the VEGFR, is highly expressed in AdCC. This increased expression correlates with stage, tumor size, vascular invasion, recurrence, and metastasis thus considering VEGFR to be the crucial receptor involved in the initiation of the formation and maintenance of tumor vasculature in AdCC [57,58,59,60]. Fibroblast growth factor (FGF) signaling is associated with the progression of AdCC. Fibroblast growth factor (FGF) 1, FGF2, and FGF receptor 1 (FGFR1) have been shown to be overexpressed in AdCC and implicated in the carcinogenesis of AdCC upregulation of extracellular signal-regulated kinase and cyclin D1 and P21 signaling pathway [61,62]. EGFR overexpression rate is seen in about 70% of salivary gland carcinoma, with a maximum of 85% in AcCC, making it an attractive therapeutic target [63,64,65]. Additionally, signaling via insulin-like growth factors (IGF)-1R has been demonstrated to play a key role in malignant transformation, anti-apoptosis, and metastatic behavior in a variety of cancers [66,67,68]. In non-small cell lung cancer, IGF-1R is often overexpressed and can mediate the cancer proliferation [69]. In addition, acquired resistance to EGFR tyrosine kinase inhibitors (TKIs) is associated with enhanced dependency on IGF-1R signaling [70,71,72,73]. Moreover, evidence is accumulating that EGFR/IGF-1R heterodimerization as well as crosstalk between the IGF-1R and EGFR pathways can induce resistance to EGFR inhibition mediated by IGF-1R and vice versa [70].

Both the androgen receptor (AR) and human epidermal growth factor receptor 2 (HER2 or ErBB2) play a role in SGMs. More particularly in SDC, as up to 90% of patients are positive for the AR, and up to 40% are positive for HER2 [13,14,15,74,75,76,77,78,79], with the rate of co-expressing AR and HER2 was found to vary between 35% and 58% [79,80]. Based on the morphologic resemblance and molecular profiling (AR expression; TP53 mutations, HRAS mutations, PIK3CA mutations, and HER2 amplification), SDC is remarkable similar to high-grade breast ductal carcinoma [75]. Next to SDC, other SGMs found to express AR include adenocarcinoma, carcinoma ex pleomorphic adenoma, MEC, and basal cell adenocarcinoma [81,82]. The biologic significance of concomitant HER2 and AR expression remains to be elucidated, although these patients seem to have a worse outcome [79,80].

Oncogenic activation of the Notch pathway, either through mutation, gene rearrangement or over-expression, is implicated in solid tumours [83,84]. Previous studies demonstrate poor prognostic outcomes in AdCC patients with NOTCH mutations [85,86].

Immune-related receptors, such as the programmed death (PD)-1/PD-Ligand (L)1 axis, have shown to play an important role in cancer homeostasis as upregulation of the PD-1 pathway leads to suppression of immune response in many tumors [87]. An analysis of 217 surgically resected SGM specimens indicated high PD-L1 expression in high-grade SGM subtypes associated with aggressive behavior (e.g., SDC). An association between PD-L1 positivity and inferior disease-free survival was also observed [88]. In addition, PD-L2 expression has been found in several tumor types, including AdCC [89,90].

#### 2.2.2. Intracellular and Epigenetic Alterations

Intracellular alterations in pathways and/or protein expression has been shown to be an important player in the carcinogenesis of SGMs.

The myeloblastosis (MYB) transcription factor regulates multiple transcriptional pathways including cellular differentiation and proliferation. Alterations and overexpression of the myeloblastosis (MYB) signaling pathway (65%) is a hallmark of AdCC [85,91]. The functional significance of this MYB overexpression is an upregulation of several growth and angiogenic factors, including VEGFA, FGF2, and c-KIT. Of note, the upregulation of VEGFA, FGF2, and c-KIT is not solely MYB-induced, suggesting dependent and independent mechanisms of VEGFR/c-KIT/PDGFR activation [92,93,94,95].

Limited data is available on the phosphatidylinositol 3-kinase (PI3K)-Akt-mammalian target of rapamycin (mTOR) pathway. Younes et al. [96], described that AdCC cell lines exhibited increased phosphorylated Akt activity when stimulated with EGF, which could be countered by treatment with an EGFR/VEGFR inhibitor. Interestingly, only the phosphorylated form of Akt decreased whereas the total level of Akt remained unchanged, indicating the importance of the phosphorylation step by PI3K in the carcinogenesis of SGMs.

NF-κB represents a family of inducible transcription factors which is involved in most gene activations. It was found that NF-κB is highly expressed in AdCC and relates to angiogenesis and poor patient outcome [60,97]. Lastly, DNA methylation and histone hypoacetylation lead to silencing of key genes, including those involved in immune recognition. Preclinical observations of various proposed mechanisms relating to altered gene expression in malignant cells, lymphocytes and cells within the tumor microenvironment support the synergistic activity of epigenetic modification and immune check-point inhibition [98].

Seen the involvement of the above-mentioned targets in SGMs, there is strong rationale for the development and testing of specific inhibitors as anti-cancer therapy in SGMs. Multiple clinical studies, of which the majority phase II studies (listed in Table 3), have been conducted in order to determine the effectiveness of these inhibitors in heterogeneous populations of SGMs [81,99,100,101,102,103,104,105,106,107,108,109,110,111,112,113,114,115,116,117,118,119,120,121,122,123,124,125,126,127,128,129,130,131,132,133,134,135,136,137,138,139,140,141,142,143,144,145].

It is however clear that, despite various specific inhibitors at our disposal, the response to therapy in advanced and metastatic SGMs remains poor.

This is especially true for the use of multiple TKIs, with most studies only indicating limit to no responses with occasional ORR reaching up to 46%. The low response to multiple TKIs can be explained by the fact that various receptors and pathways, as indicated above, can induce cancer proliferation and survival (Figure 1). As such, this multitude in pathways provide the tumor with an excellent mechanism of evading the anti-tumoral effect, induced by the TKIs. This might also explain why important differences are observed between studies using the same targeted agent. In this matter, Feeney et al. [111] reported no response to lenvatinib used in patients with AdCC whereas Locati et al. [132] and Tchekmedyian et al. [141] reported an ORR of 12% and 16%; respectively.

Use of the anti-HER2 monoclonal antibody trastuzumab on the other hand, in combination with pertuzumab (anti-HER2) or docetaxel chemotherapy showed a nice effectiveness in patients with HER2^+^ SGMs, displaying an ORR of 56% and 70%; respectively [128,140]. This reached even efficacy of 90% for use of the HER2-targeted antibody-drug conjugate ado-trastuzumab emtansine in HER2^+^ patients [129]. This illustrates the high importance for assessing the tumor pathological and molecular profile, as HER2 overexpression and alterations are typically seen in SDC. Nevertheless, this will not always be a measurement for success as the phase II study for trastuzumab monotherapy in HER2^+^ SGMs only reached an ORR of 8% [116]. It is therefore clear that SGMs attempt to induce drug resistance by activating different receptors and subsequent pathways thus rendering the anti-HER2-therapy useless, as was indicated for multiple TKIs.

The same holds true for therapies directed at the AR. Despite the strong rationale, ORR for enzalutamide (non-steroidal AR antagonist), abiraterone acetate (androgen biosynthesis inhibitors) and leuprorelin acetate + bicalutamide (gonadotropin-releasing hormone receptor agonist + non-steroidal AR antagonist) was only 15%, 21% and 42%, respectively [113,119,131]. The combination of leuprorelin acetate + bicalutamide did although result in a clinical benefit of 75% and a statistically significant increase in three-year disease-free survival (48% versus 28%).

The introduction of immune check-point inhibition (ICI) has shown modest response in treatment-resistant salivary gland cancers. One possible target is PD-L1, as its expression is associated with malignant behavior in SGMs. In a phase Ib trial, the fully humanized monoclonal anti-PD-1 therapeutic antibody pembrolizumab has been investigated during 24 months in 26 PD-L1^+^ (combined positive score ≥ 1) patients with advanced salivary gland cancers. Twelve percent of this cohort achieved a partial response while an additional 46% of patients had a stable disease course. This means that a total of 58% were classified as having disease control over 24 months [106], indicating the potential of immune checkpoint inhibitors in this patient population.

Due to the rather limited efficacy of blocking receptors involved in SGMs, it is therefore reasonable that targeting intracellular molecules, more downstream in the pathway, might harbor better effects. Although the literature on these intracellular drugs is sparse, also these drugs do not present with the highly needed efficacy for treatment of metastatic SGMs. Inhibition of mTOR, Akt, and NF-κB namely [101,121,126] showed no response in SGMs. Next, cancer stem cell-targeted systemic treatments are also now being extensively assessed. Inhibition of NOTCH signaling has generated a response rate of 0% to 17% in phase I studies [102,110,112]. Focusing more on the epigenetic alterations, by inhibiting DNA-methylation through PRMT5 inhibitors or by inhibiting histone hypoacetylation via histone deacetylase inhibitors, showed a respective ORR 21% and 9% [115,139,146]. Clinical trials utilizing Myb-targeted inhibitors are ongoing.

About outcome in the metastatic setting, if reported and defined, median PFS ranged between 2.3 months and 19.7 months, whereas median OS was between 7.0 months and 39.7 months. The substantial difference between lowest and highest reported outcomes most likely the result of which patients were included into the trials, as in most studies, number of prior systemic lines was no exclusion criteria. In addition, it is important to emphasize that the outcome is also skewed due to the heterogenous populations included into these clinical trials. In this matter, patients with metastatic AdCC or low grade adenocarcinoma have a much slower disease progression in comparison to patients with more aggressive SGMs, such as SDC and mucinous adenocarcinoma. This is clearly demonstrated by the survival data of the clinical trials with lapatinib, sorafenib, gefitinib, cabozantinib and eribulin mesylate. In these trials, the median OS for patients with AdCC is not reached, 26.4 months, 25.9 months, 27.5 months and 20.0 months, whereas OS equals 13.8 months, 12.3 months, 16.0 months, 14.2 months and 15.5 months for patients with non-AdCC; respectively. In these studies, the non-AdCC population consisted mostly from a mix of MEC, SDC, AcCC and adenocarcinoma [100,123,133,137,143].

Next, it is however also clear that highest outcomes were achieved for latest-generation TKIs (e.g., apatinib, cabozantinib, and lenvatinib) [132,141,143,145], or for combination therapies such as imatinib + cisplatin, leuprorelin acetate + bicalutamide or trastuzumab + docetaxel [113,114,140]. The latter would suggest a rational combination of targeted inhibitors of these pathways might improve patient outcome. Of course, treating physicians should be aware of the enhanced safety profile when combining these drugs to increase the success rate in salivary gland carcinoma.

So, although promising therapies have/are being investigated, which can provide the oncologist with new treatment avenues, not all aforementioned therapies are readily used as standard-of-care. For now, the unmet need for therapeutic strategies to treat advanced malignant SGMs still remains. Several clinical studies (monotherapeutic and combination therapies) are currently ongoing (Table 4). However, as we stipulated that several pathways can be used in salivary gland carcinoma to secure tumor proliferation, neo-angiogenesis and survival, the questions remain whether these trials will be successful.

It is therefore imperative that future studies not only validate the above-mentioned therapeutic efficacy findings, but also focus on the tumor biology in order to discover novel targets with higher diagnostic and/or therapeutic potential [55].

**Table 3:** Overview of targeted therapies tested in SGMs.

Phase	Setting	Agent	Target	Pts, n	Subtype *	ORR, n (%) *	Median PFS (Months)	Median OS (Months)
**A. Tyrosine kinase inhibitors**								
II [145]	R/M Any line	Apatinib	VEGFR, RET, c-KIT	65	AdCC	30 (46)	19.7	Not reached
II [118]	R/M Any line	Axitinib	VEGFR, PDGFR, c-KIT	33	AdCC	3 (9)	5.7	–
II [130]	R/M First- or second-line	Axitinib	VEGFR, PDGFR, c-KIT	26	AdCC: 6 Non-AdCC: 20 (1 SDC, 3 AcCC, 6 adeno, 2 clear cell, 1 ex pleomorphic adenoma, 1 myoepithelial, 1 epithelial-myoepithelial, 5 undifferentiated)	ITT: 2 (8) AdCC: 1 (17) Non-AdCC: 1 (5) (1 undifferentiated)	5.5	26.2
II [124]	R/M Any line	Axitinib	VEGFR, PDGFR, c-KIT	27	AdCC	0 (0)	10.8	NR
II [124]	R/M Any line	Axitinib (after corss-over)	VEGFR, PDGFR, c-KIT	26	AdCC	3 (12)	14.5	27.2
II [143]	R/M Any line	Cabozantinib	MET, RET, AXL, VEGFR2, FLT3, c-KIT.	21	AdCC: 15 Non-AdCC: 6 (1 MEC, 4 SDC, 1 AcCC, 1 ex pleomorphic adenoma)	ITT: 2 (10) AdCC: 1 (7) Non-AdCC: 1 (17) (1 SDC)	AdCC: 9.4 Non-AdCC: 7.2	AdCC: 27.5 Non-AdCC: 14.2
II [81]	R/M First-, second- or third-line	Cetuximab	EGFR	30	AdCC: 23 Non-AdCC: 7 (2 MEC, 1 AcCC, 1 adeno, 3 myoepithelial)	ITT: 0 (0) AdCC: 0 (0) Non-AdCC: 0 (0)	ITT: 6.0 AdCC: 6.0 Non-AdCC: 2.0	–
I/II [99]	Non-R/M No prior line	Cetuximab (+ IMRT)	EGFR	23	AdCC	8 (35)	NR (DFS)	54.0
II [117]	Locally advanced	Cetuximab + cisplatin	Cetuximab: EGFR Cisplatin: cytostatic drug	9	EGFR^+^ AdCC	4 (44)	64.0	Not reached
II [117]	R/M	Cetuximab + cisplatin + 5-fluorouracil	Cetuximab: EGFR Cisplatin: cytostatic drug 5-fluorouracil: cytostatic drug	12	EGFR^+^ AdCC	5 (42)	13.0	24.0
II [144]	R/M Any line	Dasatinib	c-kit, BCR-ABL, SRC family, PDGFβ, EPHA2	54	c-kit^+^ AdCC: 40 Non-AdCC: 14	ITT: 1 (2) AdCC: 1 (3) Non-AdCC: 0 (0)	AdCC: 4.8	AdCC: 14.5
II [125]	R/M Any line	Dovotinib	VEGFR, c-Kit, PDGFR, CSF-1R, RET, TrkA, FLT3	32	AdCC	1 (3)	6.0	Not reached
II [107]	R/M Any line	Dovotinib	VEGFR, c-Kit, PDGFR, CSF-1R, RET, TrkA, FLT3	34	AdCC	2 (6)	8.2	20.6
I [104]	R/M Any line	Figitumumab + dacomitinib	Figitumumab: IGF1R Dacomitinib: HER1/EGFR, HER2, HER4	5	AdCC: 3 Non-AdCC: 2	1 (20)	–	–
II [123]	R/M Any line	Gefitinib	EGFR	36	AdCC: 19 Non-AdCC: 17 (2 MEC, 3 SDC, 2 AcCC, 9 adeno, 1 myoepithelial)	ITT: 0 (0) AdCC: 0 (0) Non-AdCC: 0 (0)	AdCC: 4.3 Non-AdCC: 2.1	AdCC: 25.9 Non-AdCC: 16.0
II [122]	R/M Any line	Imatinib	c-kit, BCR-ABL, PDGFR	16	c-kit^+^ AdCC	0 (0)	2.3	7.0
II [136]	R/M Any line	Imatinib	c-kit, BCR-ABL, PDGFR	10	AdCC	0 (0)	6.0	–
Retro [103]	R/M First-line	Imatinib	c-kit, BCR-ABL, PDGFR	8	c-kit^+^ AdCC	0 (0)	3.0	–
II [114]	R/M Any line	Imatinib + cisplatin	Imatinib: c-kit, BCR-ABL, PDGFR Cisplatin: cytostatic drug	28	c-kit^+^ AdCC	3 (11)	15.0	35.0
II [100]	R/M Any line	Lapatinib	HER2, EGFR	40	EGFR^+^ or HER2^+^ AdCC: 20 Non-AdCC: 20 (2 MEC, 4 SDC, 1 AcCC, 7 adeno, 3 SCC, 3 undifferentiated)	ITT: 0 (0) AdCC: 0 (0) Non-AdCC: 0 (0)	ITT: 3.4 AdCC: 3.5 Non-AdCC: 2.1	ITT: Not reached AdCC: Not reached Non-AdCC: 13.8
II [141]	R/M Any line	Lenvatinib	VEGFR, FGFR, PDGFR, RET, KIT	32	AdCC	5 (16)	17.5	–
II [132]	R/M Any line	Lenvatinib	VEGFR, FGFR, PDGFR, RET, KIT	26	AdCC	3 (12)	9.1	27.0
Retro [111]	R/M Any line	Lenvatinib	VEGFR, FGFR, PDGFR, RET, KIT	21	AdCC	0 (0)	4.5	12.0
II [127]	R/M Any line	Nintedanib	VEGFR, FGFR, PDGFR	20	AdCC: 13 Non-AdCC: 7 (2 MEC, 1 SDC, 1 AcCC, 3 adeno)	ITT: 0 (0) AdCC: 0 (0) Non-AdCC: 0 (0)	ITT: 7.9 AdCC: 7.9 Non-AdCC: 6.3	Not reached
II [120]	R/M Any line	Regorafenib	VEGFR, FGFR, PDGFR	38	AdCC	0 (0)	–	–
II [142]	R/M Any line	Sorafenib	b-Raf, c-Raf, c- KIT, VEGFR, PDGFR, FLT3	23	AdCC	2 (11)	11.3	19.6
II [133]	R/M Any line	Sorafenib	b-Raf, c-Raf, c- KIT, VEGFR, PDGFR, FLT3	37	AdCC: 19 Non-AdCC: 18 (5 MEC, 2 SDC, 7 adeno, 3 myoepithelial, 1 undifferentiated)	ITT: 6 (16) AdCC: 2 (11) Non-AdCC: 4 (22) (1 MEC, 1 SDC, 1 adeno, 1 undifferentiated)	ITT: 5.9 AdCC: 8.9 Non-AdCC: 4.2	ITT: 23.4 AdCC: 26.4 Non-AdCC: 12.3
I [134]	R/M Any line	Sorafenib + R1507	Sorafenib: b-Raf, c-Raf, c- KIT, VEGFR, PDGFR, FLT3 R1507: IGF1R	?	AdCC	1	–	–
II [105]	R/M Any line	Sunitinib	VEGFR, c-KIT, PDGFR, RET, FLT3	13	AdCC	0 (0)	7.2	18.7
**B. Immune checkpoint inhibition**								
Ib [106]	R/M Any line	Pembrolizumab	PD-1	26	PD-L1^+^ (CPS ≥ 1) AdCC: 2 Non-AdCC: 24 (3 MEC, 1 SDC, 1 AcCC, 11 adeno, 1 myoepithelial, 1 carcinoid, 2 SCC, 1 serous, 3 undifferentiated)	ITT: 3 (12) AdCC: 0 (0) Non-AdCC: 3 (13) (2 adeno, 1 serous)	4.0	13.0
II [135]	R/M Any line	Pembrolizumab	PD-1	10	AdCC	0 (0)	6.6	27.2
II [135]	R/M Any line	Pembrolizumab (+ IMRT)	PD-1	10	AdCC	0 (0)	4.5	Not reached
II [109]	R/M Any line	Pembrolizumab	PD-1	109	AdCC: 59 Non-AdCC: 50 (4 MEC, 2 SDC, 1 AcCC, 25 adeno, 5 mucinous, 2 SCC, 3 cystadeno, 2 ex pleomorphic adenoma, 1 cystic, 2 myoepithelial, 1 epithelial-myoepithelial, 2 undifferentiated)	5 (5) PD-L1^−^ (CPS 0): 2/77 (3) PD-L1^+^ (CPS ≥ 1): 3/28 (11)	4.0	21.1
II [138]	R/M Any line	Pembrolizumab + Vorinostat	Pembrolizumab: PD-1 Vorinostat: histone deacetylase	25	AdCC: 12 Non-AdCC: 13 (3 MEC, 1 SDC, 3 AcCC, 1 adeno, 1 myoepithelial, 2 ex pleomorphic adenoma, 1 lymphoepithelioma, 1 clear cell)	ITT: 4 (16) AdCC: 1 (8) Non-AdCC: 3 (23) (2 AcCC, 1 lymphoepithelioma)	6.9	14.0
**C. HER2-targeted therapy**								
II [116]	R/M First-, second- or third-line	Trastazumab	HER2	14	HER2^+^ AdCC: 2 Non-AdCC: 12 (3 MEC, 7 adeno, 2 SCC)	AdCC: 0 (0) Non-AdCC: 1 (8) (1 MEC)	4.2	–
II [128]	R/M Any line	Trastuzumab + Pertuzumab	Trastuzumab: HER2 Pertuzumab: HER2	16	HER2^+^ non-AdCC (1 MEC, 3 SDC, 8 adeno, 4 unspecified)	9 (56)	9.1	20.4
II [140]	R/M Any line	Trastuzumab + Docetaxel	Trastuzumab: HER2 Docetaxel: cytostatic drug	57	HER2^+^ SDC	40 (70)	8.9	39.7
II [129]	R/M Any line	Ado-trastuzumab emtansine	HER2-targeted ADC	10	HER2^+^	9 (90)	Not reached	Not reached
**D. AR-targeted therapy**								
II [119]	R/M Any line	Enzalutamide	AR	46	AR^+^ AdCC:1 Non-AdCC: 45 (41 SDC, 1 adeno, 3 ex pleomorphic adenoma)	7 (15)	5.6	17.0
II [131]	R/M Any line	Abiraterone acetate	CYP17A1	24	AR^+^ non-AdCC (19 SDC, 5 adeno)	5 (21)	ITT: 3.7 SDC: 4.0 Adeno: 2.5	ITT: 22.5 SDC: Not reached Adeno: 8.8
II [113]	R/M Any line	Leuprorelin acetate + Bicalutamide	Leuprorelin acetate: GnRH receptor agonist Bicalutamide: AR	36	AR^+^ non-AdCC (34 SDC, 2 adeno)	15 (42)	8.8	30.5
**E. Notch**								
I [112]	R/M Any line	Brontictuzumab	NOTCH1	12	AdCC	2 (17)	–	–
I [102]	R/M Any line	BMS-986115	pan-NOTCH	5	AdCC	0 (0)	–	–
I [110]	R/M Any line	Crenigacestat	pan-NOTCH	22	AdCC	1 (5)	ITT: 5.3 1st-line: Not reached 2nd-line: 7.7 3rd+-line: 2.4	–
**F. Other therpies**								
II [121]	R/M Any line	Nelfinavir	Akt pathway	15	AdCC	0 (0)	5.5	–
II [126]	R/M Any line	Everolimus	mTOR	34	AdCC	0 (0)	11.2	23.7
II [101]	R/M Any line	Bortezomib	NF-κB	21	AdCC	0 (0)	6.4	21.0
II [101]	R/M Any line	Bortezomib + doxorubcin	Bortezomib: NF-κB Doxorubcin: cytostatic drug	10	AdCC	1 (10)	6.4	21.0
II [137]	R/M Any line	Eribulin mesylate	Microtubule inhibitor	29	AdCC: 11 Non-AdCC: 18 (2 MEC, 1 SDC, 2 AcCC, 4 adeno, 3 myoepithelial, 2 ex pleomorphic adenoma, 1 mammary analogue, 1 clear cell, 2 undifferentiated)	ITT: 3 (10) AdCC: 2 (18) Non-AdCC: 1 (6) (1 ex pleomorphic adenoma)	ITT: 3.5 AdCC: 3.5 Non-AdCC: 3.3	ITT: 16.0 AdCC: 20.0 Non-AdCC: 15.5
I [139]	R/M Any line	GSK3326595	PRMT5	14	AdCC	3 (21)	–	–
I [146]	R/M Any line	Chidamide	Histone deacetylase	3	Undefined	1 (33)	–	–
II [115]	R/M Any line	Vorinostat	Histone deacetylase	30	AdCC	2 (7)	10.0	11.5

* If specified in the original study, the subtyping of the non-AdCC tumors is given. AcCC, acinic cell carcinoma; ADC, antibody-drug conjugate; AdCC, adenoid cystic carcinoma; AR, androgen receptor; BCR-ABL, breakpoint cluster region-Abelson; CPS, combined positive score; CSF-1R, colony stimulating factor-1 receptor; CYP17A1, 17α-hydroxylase; DFS, disease-free survival; EGFR, epidermal growth factor receptor; EPHA2, Ephrin A2; FGFR, fibroblast growth factor receptor; FLT3, fms-like tyrosine kinase 3; GnRH, gonadotropin hormone-releasing hormone; HER, human epidermal growth factor receptor; IGF1R, insulin-like growth factor 1 receptor; ITT, intention-to-treat; MEC, mucoepidermoid carcinoma; MET, hepatocyte growth factor receptor; mTOR, mammalian target of rapamycin; n, number; NF-κB, nuclear factor-κB; NOTCH, Neurogenic locus notch homolog protein; ORR, objective response rate; OS, overall survival; PD-1, programmed cell death-1; PDGFR, platelet-derived growth factor receptor; PDGF, platelet-derived growth factor; PD-L1, programmed cell death-ligand 1; PFS, progression-free survival; PRMT5, protein arginine methyltransferase 5; Pts, patients; R/M, recurrent or metastatic; RET, rearranged during transfection; Retro, retrospective; SCC, squamous cell carcinoma; SDC, salivary duct carcinoma; SGMs, salivary gland malignancies; SRC, steroid receptor coactivator; TrkA, tropomyosin receptor kinase A; VEGFR, vascular endothelial growth factor receptor.

**Table 4:** Ongoing clinical trials in SGMs.

Phase	Trial Identification	Setting	Agent	Target	Pts, n	Subtype
I	NCT03886831	R/M-Any line	PRT543	PRMT5	227	AdCC (+other indications)
I	NCT03291002	R/M-Any line	CV8102 + anti-PD-1 therapy	VMD-928: TLR7/8 agonist anti-PD-1 therapy: PD-1	98	AdCC (+other indications)
I	NCT03556228	R/M-Any line	VMD-928	VMD-928: TrkA	74	AdCC (+other indications)
I	NCT03287427	R/M-Any line	TetMYB vaccine + tislelizumab	TetMYB vaccine: cancer therapy vaccine Tislelizumab: PD-1	32	AdCC (+CRC)
II	NCT03999684	R/M-Any line	Tretinoin	retinoic acid receptor	27	AdCC
II	NCT03691207	R/M-Any line	AL101	pan-NOTCH	87	AdCC
II	NCT03422679	LA or R/M-Any line	CB-103	pan-NOTCH	200	AdCC (+other indications)
I/II	NCT03781986	R/M-Any line	APG-115 ± carboplatin	APG-115: MDM2 Carboplatin: cytostatic drug	32	AdCC Non-AdCC
II	NCT03639168	R/M-Any line	chidamide + cisplatin	chidamide: histone deacetylase cisplatin: cytostatic drug	22	AdCC
II	NCT04209660	R/M-Any line	Lenvatinib + pembrolizumab	Lenvatinib: VEGFR, FGFR, PDGFR, RET, KIT Pembrolizumab: PD-1	64	AdCC Non-AdCC
II	NCT04119453	R/M-Any line	Apatinib	VEGFR, RET, c-KIT	80	AdCC

This list is not comprehensive. AdCC, adenoid cystic carcinoma; FGFR, fibroblast growth factor receptor; LA, locally advanced; NOTCH, Neurogenic locus notch homolog protein; PD-1, programmed cell death-1; PDGFR, platelet-derived growth factor receptor; PRMT5, protein arginine methyltransferase 5; Pts, patients; R/M, recurrent or metastatic; RET, rearranged during transfection; TrkA, tropomyosin receptor kinase A; SGMs, salivary gland malignancies; VEGFR, vascular endothelial growth factor receptor.

## 3. Novel Targets and Biomarkers

Overall, promising research is being undertaken to further unravel the etiology of SGMs by focusing o.a. on specific protein targets and molecular pathways.

Firstly, prostate-specific membrane antigen (PSMA), a type II transmembrane glycoprotein, is of high interest in SGMs. Three studies explored PSMA-targeted PET/CT performance in SGMs, majority being AdCC [147,148,149]. The only prospective study showed an added diagnostic value for PSMA radioligand PET/CT in 4 out of the 15 AdCC patients; for identification of bone metastases (2), an AdCC local recurrence in the Bartholin gland (1) and additional lymph node metastases (1). Further investigations are ongoing.

Secondly, molecular profiling of the tumor is becoming of increasing interest for all solid malignancy. Recently, decreased expression of c-kit, an epithelial to mesenchymal transition-related protein has been found in MEC. These proteins promote invasion and metastasis. Conversely, c-kit expression was associated with perineural infiltration and tumor grade in AdCC [56,150]. Pan-cancer analysis has shown an activating IGF-1R hotspot non-frameshift insertions, which were significantly enriched in AdCC. This may represent a novel subtype of AdCC which may be more susceptible to existing IGF1R inhibitors [151]. Next generation sequencing targeting NOTCH1/2/3 genes and/or NOTCH1 intra-cellular domain (NICD1) has indicated that patients with NOTCH pathway activated AdCC have worse clinical outcomes [152,153]. Novel treatment options, combined with Notch inhibitors, are therefore needed in this clinically poor group. In addition, multiple research groups are focusing on molecular abnormalities (DNA as well as RNA) which may be of use for SGMs [154,155,156,157,158,159,160]. Selecting patients based on the tumor molecular profile can be considered a major advantage and should be recommended as this can guide the development of novel therapeutic strategies according to disease biology and further personalize patient treatment. On top of this, several fusion genes are known for their involvement in salivary gland carcinoma. It is known that MYB/MYBL1-NFIB fusions are being detected in less than 60% of all AdCC [94], remaining 40% of AdCC without clear oncogenic driver mutations. Recently, Shibata et al. [161] identified six new gene fusions. Of these, NFIB-EPB41L2 and NFIB-MCMDC2 are considered to activate MYB and MYBL1 expression, thus involved in the carcinogenesis of AdCC. These targets may prove useful as potential future target for treatment of this malignancy. Comparably for MEC, it has been shown that the CRTC1/MAML2 fusion is present in up to 56% of cases [162]. Furthermore, earlier research has demonstrated that the CRTC1/MAML2 fusion is necessary for MEC cell proliferation and survival through stimulation of the EGFR signaling pathway [163,164]. In addition, Ni et al. [165] identified the critical role of Notch signaling in maintaining MEC stem-like cells and tumor growth, suggesting that co-targeting of Notch and EGFR might be a potential effective anti-MEC treatment.

Thirdly, we have already indicated that several pathways can be used by the tumor to maintain the homeostasis. Recent genetic analysis has shown that upregulation of hypoxia-inducible factor 1α (HIF-1α) upregulation enhances transcriptional activity and expression of nidogen 1, which subsequently promotes AdCC metastasis via PI3K/Akt pathway activation [166]. Comparably, Sphk1 is overexpressed in AdCC and promotes salivary tumorigenesis by activating the PI3K/Akt pathway [167]. On the other hand, the type III TGF-β receptor is significantly decreased in AdCC, allowing for NF-κB to induced cell proliferation and migration [168]. Therefore, Nidogen 1, Sphk1 and the type III TGF-β receptor could therefore serve as a novel theragnostic in AdCC, potentially in combination with an Akt- or NF-κB-inhibitor; respectively.

Fourthly, only a small proportion of patients seem to profit from ICI with the know anti PD-(L)1 inhibitors. Recently, it has been shown that activation of adenosine-signaling can result in a tolerogenic protumoral microenvironment. [169,170,171]. CD39 and CD73, two adenosine-signaling pathway markers are shown to be present in up to 48.2% and 42.9%, respectively [172]. This adenosine-signaling signature can therefore play a decisive role in tumor immunity and presents itself as a promising therapeutic option for patients with an activated adenosine-signaling pathway. Next, in their review, Witte et al. [160] have provided a nice summary of the current knowledge on the TME in SGMs (according to entity). This can further guide the development of novel (immune-) targeted therapeutic strategies per patient group. This has also been highlighted by Alame et al. [173] who stipulate that ICI or even macrophage- or NK cell-directed therapy can be highly beneficial in immune-infiltrated SDC whereas the focus should lie on molecular targets in immune-poor SDC.

Fifthly, also proteomics can provide us with some interesting targets in case of SGMs. Several proteins have been revealed to potentially aid in the diagnosis and prognosis of SGMs in a systematic review of mass spectrometry-based proteomics [174]. Annexins (ANXA) are a family of calcium-dependent, phospholipid-binding proteins that play a role in cell growth, inflammation, and signaling. Annexins A1 and A4 have been linked to benign tumors, whilst ANXA5 has been linked to malignant salivary tumors. Furthermore, ANXA5 is related with progression, invasion, and metastasis in malignant salivary gland cancers. CRYAB are small heat shock proteins that prevent denatured proteins from aggregating and sustain apoptosis and inflammation in diseases. Salivary gland cancers have been found to exhibit positive regulation of these proteins. In addition, this protein has also been found in other cancers such as breast cancer, lung cancer, prostate cancer, and ovarian cancer. It serves as an anti-apoptotic protein that downregulates the proapoptotic signaling molecules Bcl-2, Bax and caspase 3 [175]. Fibrinogen beta chain (FGB), a component of fibrinogen, is a biomarker for hepatic metastatic colorectal cancer. This protein has found to be overexpressed in malignant salivary tumors and is linked to tumor growth, invasion, and metastasis, whereas reduced expression is more prevalent in benign tumors. GNB2L1 is an intracellular scaffold protein that assists in cell growth, migration, and differentiation. Pleomorphic adenoma as well as malignant SGMs appear to have an overexpression of GNB2L1, and tis protein might therefore be used as a diagnostic marker. The glycoprotein IGHG1, which is produced by B lymphocytes, was shown to be more abundant in benign Warthin tumors and downregulated in malignant SGMs.

Despite these encouraging results, there is a lack of uniformity of proteomics data as well as the inability to determine the number of proteins in each lesion. Thus, further in-depth evaluation is highly recommended to use these proteins in a diagnostic fashion or as potential target in novel systemic therapies.

Based on this overview, various research groups are looking for ways to enhance the efficacy of the therapeutic options administered in salivary gland carcinoma. Although the full etiology remains to be elaborated, it is becoming more obvious that a deeper characterization of each separate case is needed to maximize and personalize patient care.

## 4. Case Description

To illustrate the high need for a more personalized approach, by use of molecular diagnostics and novel treatment targets, we provide the following case from our university center.

In November 2017, a 57-year-old patient presented with sudden peripheral paralysis of the left facial nerve of the left side on the emergency department. The patient recalled an infection with cutaneous Herpes Zoster some weeks before. An MRI of the brain observed hypercaptation of the left facial nerve in the tympanic and mastoid segment. Altogether, results indicated diagnosis of Bell’s palsy. The patient was sequentially treated with corticosteroids, electrostimulation, mime therapy and massage-therapy but no significant clinical improvement was noticed.

In August 2018, the patients’ general practitioner noticed a painless swelling and retraction of the left retroauricular region on which a CT scan was done in our institution. A necrotic, tumor mass originating from the deep lobe of the left parotic gland infiltrating the skin and facial nerve up to the stylomastoid foramen was discovered. Bilateral cervical lymph node involvement was observed. A superficial and deep biopsy revealed presence of an invasive high-grade salivary duct adenocarcinoma originating from the parotic gland with lymphovascular and perineural invasion. The immunohistochemical profile showed Cytokeratin 7 expression and diffuse androgen receptor (AR) expression, of which additional progesterone and estrogen receptors were found both negative. Her2Neu immune expression was 2+ but without amplification covered by FISH (ratio HER2/CEP17 1.42) on 20 invasive cell cores. NTRK-gene fusion was absent.

Multidisciplinary oncology consultation concluded a stage cT4a cN3b cM0. As surgical treatment was considered unfeasible, the patient was alternatively treated with primary radiotherapy combined with maximal androgene blockade of bicalutamide (Casodex^®^) 50 mg PO QD + leuproreline injection (Depo-Eligard^®^) 7.5 mg QM). A total dose of 69.12 Gy on the primary tumor and 56 Gy on the elective neck in 32 fractions was administered with curative intent. PET-CT scan viewed a regression in volume and metabolic activity of primary tumor and lymph nodes, concluding a partial remission. As tolerability to androgen blockers was unfavorable, bicalutamide (Casodex^®^) and leuproreline injection (Depo-Eligard^®^) were temporarily ceased and the patient was subsequently treated with adjuvant chemotherapy, Paclitaxel (Taxol^®^) 80 mg/m^2^ IV Q1W combined with trastuzumab (Herceptine^®^) 600 mg IV Q3W. Again, partial remission was attained after four cycles with an acceptable tolerance profile. Current treatment regimen was continued for another four cycles. During treatment, the patient was diagnosed with a treatment-related venous thromboembolic event with lung embolism infarction (for which low-molecular-weight heparins were initiated) but also experienced a significant recuperation of the facial nerve function. After eight cycles of therapy, stable disease was reached, and the patient was treated with trastuzumab as maintenance therapy. The patient remained in remission from July 2019 until May 2020, as progressive disease of the primary tumor was again observed. An in-house NGS sequencing panel of 69 genes was performed, revealing TP53 and PTEN mutations, and CDK4 amplification. The latter was considered an interesting target-based therapeutic approach and after several round-table discussions with clinicians and patient we decided to commence treatment with CDK4/6 inhibitor, palbociclib (Ibrands^®^) 125 mg PO QD combined with a reintroduction of bicalutamide (Casodex^®^) 50 mg PO QD. Patient shows excellent tolerability to the combination therapy. After over 80 weeks of therapy, patient presented with intermittent paresthesia in the left retroauricular region, probably due to a discrete growth of the salivary duct adenocarcinoma along the facial nerve. There was no evidence of distant metastases. Patient received methylprednisolone 64 mg QD PO for the intermittent paresthesia and patient was rechallenged, due to the successfulness in the past, to Paclitaxel (Taxol^®^) 80 mg/m^2^ IV Q1W combined with trastuzumab (Herceptine^®^) 600 mg IV Q3W in April 2022. However, imaging after 3 cycles of therapy indicated progressive disease with a mass increase in the left parotid lodge, further perineural invasion into the facial and auriculotemporal nerve and local lymph node involvement. Next, patient was referred for treatment with ^177^Lutetium-PSMA, although therapy was not administered due to an absent PSMA expression on the SDC tissue samples. Subsequently, patient recently started with Trastuzumab-Emtansine (Kadcyla^®^) 3.6 mg/kg IV Q3W, an antibody drug conjugate which shows excellent response in patients with HER2^+^ SDC, especially when treated previously with trastuzumab (Herceptine^®^). At current time, patient has received 2 cycles and shows only limited toxicity to the therapy (grade I AST, ALT and LDH increase).

## 5. Conclusions

Treatment options for advanced SGMs are currently extensively being investigated, and pathological and therapeutic progress is being made. If surgery is considered unfeasible, radiotherapy and chemotherapy are currently the standard of care. These treatments, however, are not effective in case of recurrent or metastatic disease, and the medical oncologist is left with limited treatment options. Based on published trials, drugs based on the expression of specific molecular markers may give extra disease control. Nevertheless, data from large clinical studies are lacking, therefore leaving the question open which is the most appropriate treatment option in SGMs. By means of our case report, we have clearly showed that research into novel genetic alternations as well as combination therapies in these cancers is highly recommended achieving the best possible disease control. Selecting patients based on their molecular profile should be endorsed to guide patient stratification as well as the development of novel therapeutic strategies. This especially as we pointed out that various pathways can be used by the tumor to create resistance to the specific therapies administered. This developed drug-resistance implies that timely examinations, f.i. through liquid biopsies, are a necessity in SGMs to improve patient follow-up. This will allow the medical oncologist to switch therapy based on the patient’s molecular profile, even before progression has been detected.

## Figures and Tables

**Table 2:** Overview of chemotherapies tested in SGMs.

Study	Setting	Agent	Target	Pts, n	Subtype *	ORR, n (%) *	Median PFS (Months)	Median OS (Months)
**A. Monotherapy**								
Retro [50]	R/M Any line	5-fluorouracil	cytostatic drug	12	AdCC	4 (33)	–	ITT: 21.0
II [49]	R/M Any line	Cisplatin	cytostatic drug	10	AdCC	7 (70)	–	–
II [46]	R/M First- or second-line	Cisplatin	cytostatic drug	25	AdCC: 13 Non-AdCC: 12 (5 MEC, 5 adeno, 1 AcCC, 1 SCC)	ITT: 4 (16) AdCC: 2 (15) Non-AdCC: 2 (17) (1 MEC, 1 SCC)	7.0	14.0
II [37]	R/M First- or second-line	Cisplatin	cytostatic drug	10	AdCC	0 (0)	3.0	21.0
Retro [36]	R/M First-line	Cisplatin	cytostatic drugs	34	AdCC: 11 Non-AdCC: 23 (7 MEC, 14 adeno, 2 mixed)	ITT: 13 (38) AdCC: 2 (18) Non-AdCC: 11 (48) (3 MEC, 7 adeno, 1 mixed)	7.0	15.0
II [53]	R/M First- or second-line	Epirubicin	cytostatic drug	20	AdCC	2 (10)	3.7	15.6
II [52]	R/M First-line	Gemcitabine	cytostatic drug	21	AdCC	0 (0)	–	–
Retro [50]	R/M Any line	Methotrexate	cytostatic drug	7	AdCC	0 (0)	–	ITT: 21.0
II [54]	R/M First-line	Mitoxantrone	cytostatic drug	32	AdCC	4 (13)	–	18.0
II [47]	R/M Any line	Mitoxantrone	cytostatic drug	18	AdCC	1 (5)	–	19.0
II [40]	R/M First-line	Paclitaxel	cytostatic drug	45	AdCC: 14 Non-AdCC: 31 (14 MEC, 17 adeno)	ITT: 8 (18) AdCC: 0 (0) Non-AdCC: 8 (26) (3 MEC, 5 adeno)	4.0	ITT: 12.5
II [34]	R/M First- or second line	Vinorelbine	cytostatic drug	20	AdCC: 13 Non-AdCC: 7 (5 adeno, 1 mixed, 1 undifferentiated carcinoma)	ITT: 4 (20) AdCC: 2 (15) Non-AdCC: 2 (29) (2 adeno)	5.0	8.5
**B. Combination therapy**								
II [32]	R/M Any line	Carboplatin + paclitaxel	cytostatic drugs	14	AdCC: 10 Non-AdCC: 9 (1 MEC, 1 adeno, 2 undifferentiated)	ITT: 2 (14) AdCC: 2 (20) Non-AdCC: 0 (0)	6.0–13.5	12.5
Retro [48]	R/M Any line	Carboplatin + paclitaxel	cytostatic drugs	38	AdCC: 9 Non-AdCC: 29 (1 MEC, 18 SDC, 4 adeno, 1 myoepithelial, 1 epithelial-myoepithelial, 4 ex pleomorphic adenoma)	ITT: 15 (39) AdCC: 1 (9) Non-AdCC: 14 (48)	ITT: 6.5 AdCC: 9.7 Non-AdCC: 6.2–6.5	ITT: 26.5 AdCC: 21.9 Non-AdCC: 34.7–44.0
II [41]	R/M Any line	Cisplatin + 5-fluorouracil	cytostatic drugs	11	AdCC	0 (0)	9.0	12.0
II [37]	R/M First- or second line	Cisplatin + doxorubicin + bleomycin	cytostatic drugs	9	AdCC	3 (33)	10.0	12.0
Retro [39]	R/M Any line	Cisplatin + doxorubicin + cyclophosphamide	cytostatic drugs	13	AdCC: 9 Non-AdCC: 4 (4 adeno)	ITT: 6 (46) AdCC: 3 (33) Non-AdCC: 3 (75) (3 adeno)	–	–
Retro [35]	R/M Any line	Cisplatin + doxorubicin + cyclophosphamide	cytostatic drugs	8	AdCC: 4 Non-AdCC: 4 (3 MEC, 1 adeno)	ITT: 5 (63) AdCC: 1 (25) Non-AdCC: 4 (100) (3 MEC, 1 adeno)	5.0	11.0
II [45]	R/M Any line	Cisplatin + doxorubicin + cyclophosphamide	cytostatic drugs	22	AdCC: 12 Non-AdCC: 10 (1 MEC, 2 SDC, 2 adeno, 1 NET, 3 myoepithelioma, 1 undifferentiated)	ITT: 6 (27) AdCC: 3 (25) Non-AdCC: 3 (30) (1 MEC, 1 SDC, 1 NET)	–	21.0
II [38]	R/M First-line	Cisplatin + doxorubicin + cyclophosphamide + 5-fluorouracil	cytostatic drugs	16	AdCC: 7 Non-AdCC: 9 (1 MEC, 8 adeno)	ITT: 8 (50) AdCC: 3 (42) Non-AdCC: 5 (56) (1 MEC, 4 adeno)	–	16.8
II [34]	R/M First- or second line	Cisplatin + vinorelbine	cytostatic drugs	16	AdCC: 9 Non-AdCC: 7 (1 MEC, 4 adeno, 2 undifferentiated)	ITT: 7 (44) AdCC: 4 (44) Non-AdCC: 3 (43) (2 adeno, 1 undifferentiated)	7.0	11.0
II [33]	R/M First-line	Cisplatin + vinorelvine	cytostatic drugs	42	AdCC: 24 Non-AdCC: 18 (2 MEC, 11 adeno, 2 mixed, 3 undifferentiated)	ITT: 13 (31) AdCC: 7 (17) Non-AdCC: 7 (39) (7 adeno)	6.0	10.0
II [33]	R/M Second-line	Cisplatin + vinorelvine	cytostatic drugs	18	AdCC: 10 Non-AdCC: 8 (9 MEC, 4 adeno, 2 undifferentiated)	ITT: 1 (6) AdCC: 0 (0) Non-AdCC: 1 (13) (1 adeno)	3.5	4.0
II [42]	R/M Any line	Cisplatin + vinorelvine	cytostatic drugs	40	AdCC: 19 Non-AdCC: 21 (6 MEC, 10 adeno, 2 AcCC, 1 myoepithelial, 1 ex pleomorphic adenoma, 1 undifferentiated)	ITT: 14 (35) AdCC: 6 (32) Non-AdCC: 8 (38)	6.3	16.9
II [51]	R/M First-line	Cyclophosphamide + vincristine + 5-fluorouracil	cytostatic drugs	8	AdCC	2 (25)	28.0	62.0
II [43]	R/M First-line	Platin + docetaxel	cytostatic drugs	41	AdCC: 26 Non-AdCC: 15 (1 MEC, 10 SDC, 3 adeno, 1 SCC)	ITT: 19 (46) AdCC: 6 (23) Non-AdCC: 13 (87) (1 MEC, 9 SDC, 2 adeno, 1 SCC	ITT: 9.4 AdCC: 8.9 Non-AdCC: 10.5	ITT: 28.2 AdCC: 27.6 Non-AdCC: 29.3
II [44]	R/M First- or second line	Platin + gemcitabine	cytostatic drugs	33	AdCC: 10 Non-AdCC: 23 (4 MEC, 1 SDC, 5 AcCC, 9 adeno, 4 undifferentiated)	ITT: 8 (27) AdCC: 2 (20) Non-AdCC: 6 (26) (1 MEC, 1 SDC, 1 AcCC, 3 adeno)	–	13.8

* If specified in the original study, the subtyping of the non-AdCC tumors is given. AcCC, acinic cell carcinoma; AdCC, adenoid cystic carcinoma; ITT, intention-to-treat; MEC, mucoepidermoid carcinoma; n, number; ORR, objective response rate; OS, overall survival; Pts, patients; PFS, progression-free survival; R/M, recurrent or metastatic; retro, retrospective; SCC, squamous cell carcinoma; SDC, salivary duct carcinoma; SGMs, salivary gland malignancies.

## Data Availability

Not applicable.
